# Renin–angiotensin–aldosterone pathway modulators in chronic kidney disease: A comparative review

**DOI:** 10.3389/fphar.2023.1101068

**Published:** 2023-02-13

**Authors:** Saeed Alshahrani

**Affiliations:** Department of Pharmacology and Toxicology, College of Pharmacy, Jazan University, Jizan, Saudi Arabia

**Keywords:** hypertension, aliskiren, angiotensin-converting enzyme inhibitors, angiotensin II receptor blockers, chronic kidney disease

## Abstract

Chronic kidney disease presents a health challenge that has a complex underlying pathophysiology, both acquired and inherited. The pharmacotherapeutic treatment options available today lower the progression of the disease and improve the quality of life but cannot completely cure it. This poses a challenge to the healthcare provider to choose, from the available options, the best way to manage the disease as per the presentation of the patient. As of now, the recommended first line of treatment to control the blood pressure in chronic kidney disease is the administration of renin–angiotensin–aldosterone system modulators. These are represented mainly by the direct renin inhibitor, angiotensin-converting enzyme inhibitors, and angiotensin II receptor blockers. These modulators are varied in their structure and mechanisms of action, hence showing varying treatment outcomes. The choice of administration of these modulators is determined by the presentation and the co-morbidities of the patient, the availability and affordability of the treatment option, and the expertise of the healthcare provider. A direct head-to-head comparison between these significant renin–angiotensin–aldosterone system modulators is lacking, which can benefit healthcare providers and researchers. In this review, a comparison has been drawn between the direct renin inhibitor (aliskiren), angiotensin-converting enzyme inhibitors, and angiotensin II receptor blockers. This can be of significance for healthcare providers and researchers to find the particular loci of interest, either in structure or mechanism, and to intervene as per the case presentation to obtain the best possible treatment option.

## 1 Introduction

Chronic kidney disease (CKD) is a persistent health condition characterized by progressive renal dysfunction that leads to a permanent and irremediable loss of renal function (Saucedo et al., 2018; Vaidya and Aeddula, 2022). CKD affects nearly 840 million people worldwide and is anticipated to become the world’s fifth major cause of mortality by 2040 (Foreman et al., 2018; Jager et al., 2019). CKD manifests as abnormalities of renal structure or function that are present for 3 months or more, with an approximate glomerular filtration rate of <60 mL/min/1.73 m^2^ and presents varied adverse health outcomes. The classification of CKD is performed as per the underlying cause, glomerular filtration rate (GFR), and category of albuminuria (Table 1) (Cheung et al., 2021; Fish et al., 2021). The condition is more often associated with aging; however, it is not uncommon in younger age groups and is a well-recognized basis for high morbidity and mortality worldwide, especially in patients with diabetes and hypertension (Kalantar-Zadeh et al., 2021). The condition progresses with no notable symptoms, hindering an early diagnosis and consequently leading to worsened treatment outcomes. A lack of sensitive and specific biomarkers for identification of the disease at early stages often leads to detection at an intermediate or late stage, when the life expectancy is already compromised and the treatment challenged (Saucedo et al., 2018). The etiology of CKD shows considerable global variation, but its cause is still debatable. However, a classical division of the renal injuries leading to CKD is prerenal, intrinsically renal, or postrenal, and the most common primary diseases that are strongly associated with the development of CKD are diabetes, hypertension, glomerulonephritis, and cystic kidney diseases. In addition, any severe or long-standing kidney injury may lead to CKD (Kalantar-Zadeh et al., 2021).

**TABLE 1:** Classification of CKD according to glomerular filtration rate (GFR), albuminuria category, and the target blood pressure as per NICE and KDIGO.

			Persistent albuminuria categories			Target BP	
			ACR category A1: (lesser than 3 mg/mmol)	ACR category A2: (3–30 mg/mmol)	ACR category A3: (more than 30 mg/mmol)		
			Normal to mildly increased	Moderately increased	Severely increased	Target BP (as per KDIGO, 2021)	Target BP (as per NICE, 2021)
GFR categories	GFR category G1: normal and high (90 mL/min/1.73 m^2^ or over)	Normal or high	Minimal risk. No CKD if no other markers of kidney damage are present	Modest risk	High risk	<120/80 mmHg (if tolerated)	<130/80 mmHg (if tolerated)
	GFR category G2: (60–89 mL/min/1.73 m^2^)	Slightly decreased	Minimal risk. No CKD if no other markers of kidney damage are present	Modest risk	High risk	<120/80 mmHg (if tolerated)	<130/80 mmHg (if tolerated)
	GFR category G3a: (45–59 mL/min/1.73 m^2^)	Slightly to modestly decreased	Modest risk	High risk	Very high risk	<120/80 mmHg (if tolerated)	<130/80 mmHg (if tolerated)
	GFR category G3b: (30–44 mL/min/1.73 m^2^)	Modestly to severely decreased	High risk	Very high risk	Very high risk	<120/80 mmHg (if tolerated)	<130/80 mmHg (if tolerated)
	GFR category G4: (15–29 mL/min/1.73 m^2^)	Severely decreased	Very high risk	Very high risk	Very high risk	<120/80 mmHg (if tolerated)	<130/80 mmHg (if tolerated)
	GFR category G5: (under 15 mL/min/1.73 m^2^)	Kidney failure (requires transplant)	Very high risk	Very high risk	Very high risk	<120/80 mmHg if tolerated, without dialysis or transplant. <130/80 mmHg if tolerated, with dialysis or transplant	<130/80 mmHg if tolerated

**FIGURE 1:**
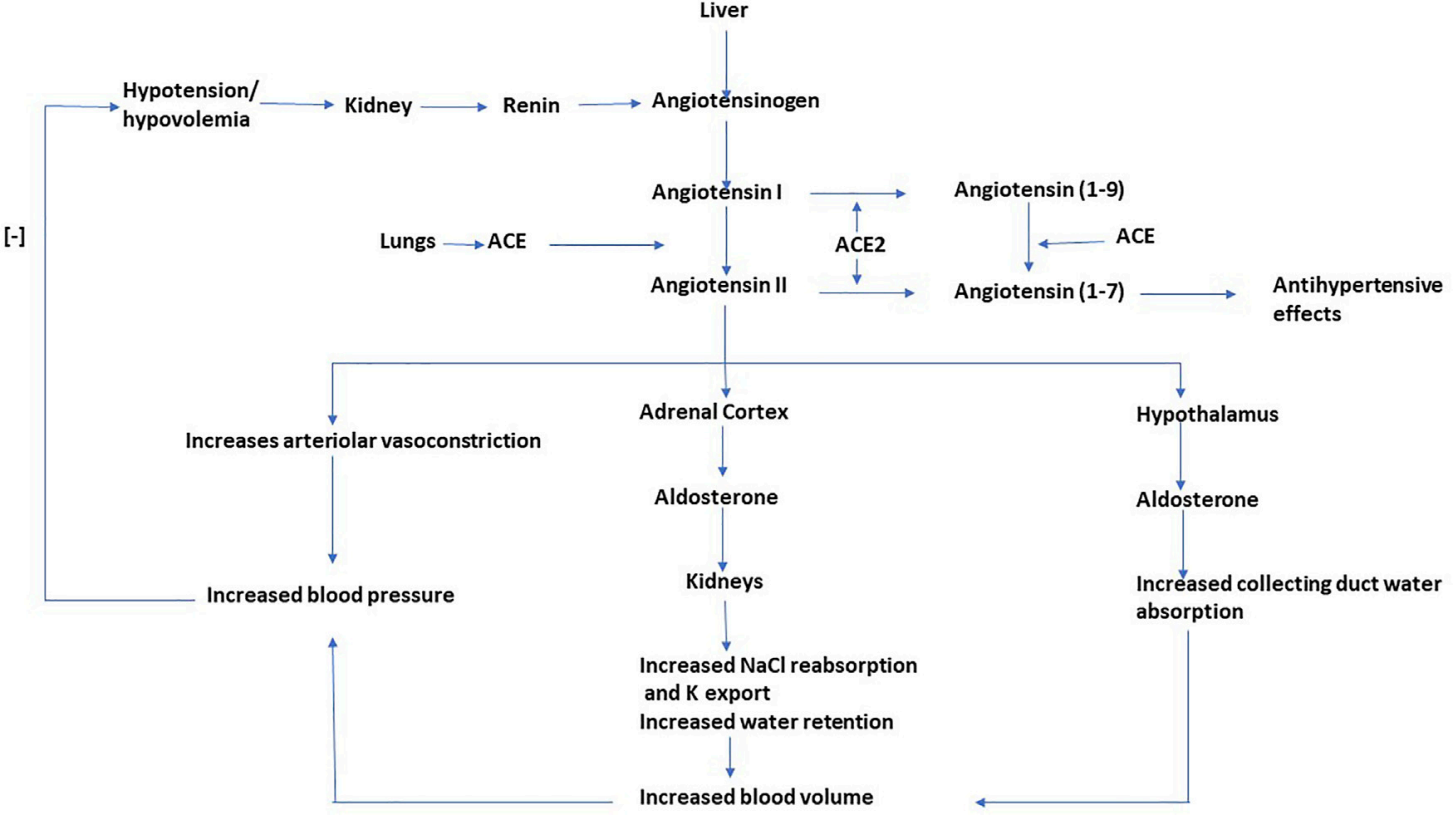
The role of RAAS in maintaining blood volume and blood pressure.

**FIGURE 2:**
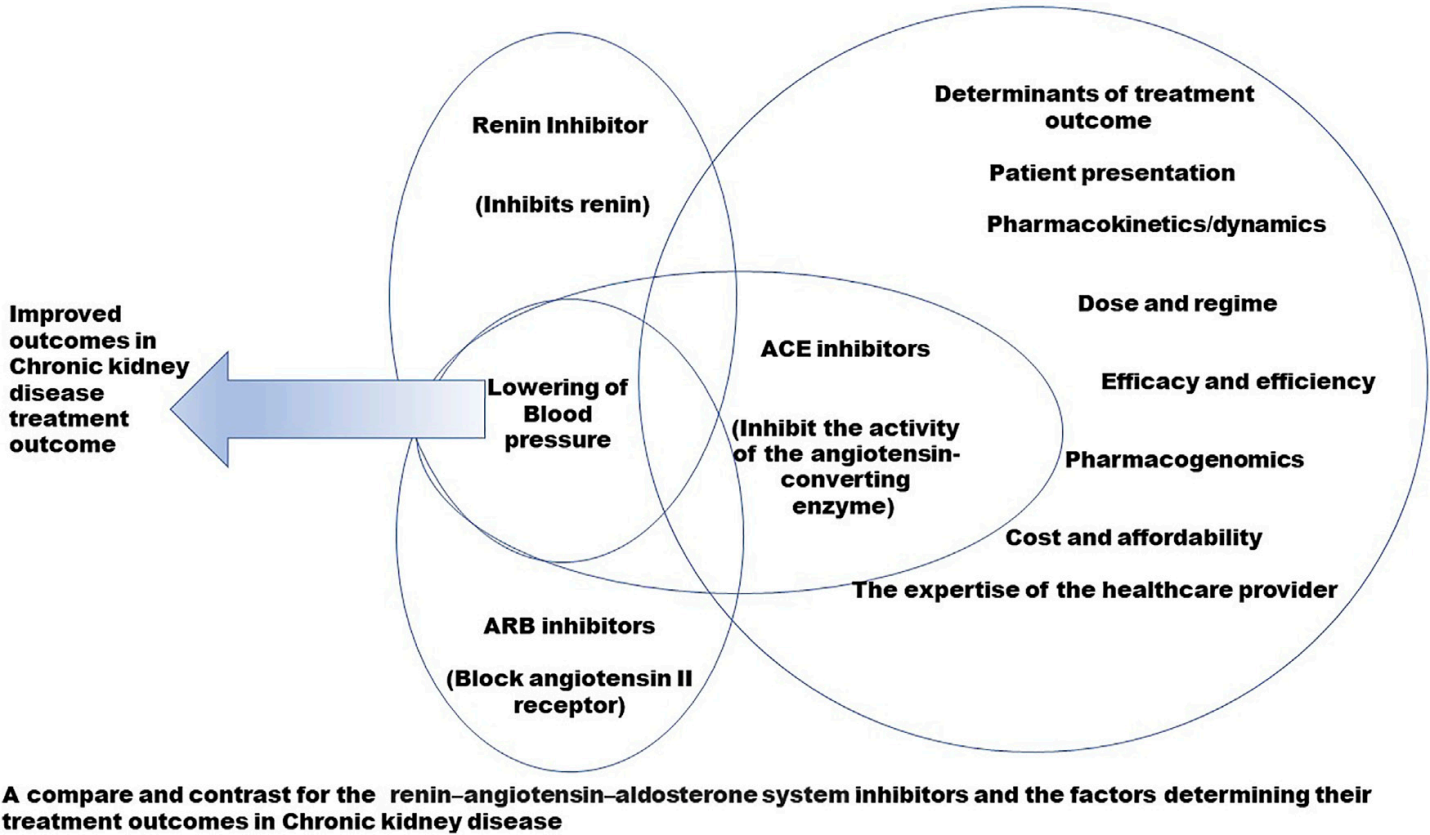
Graphical summary of the study.

Hypertension (HTN) is a critical contributor to the pathophysiology of CKD (27.2%), with HTN and CKD implicated in a cyclical manner (Vaidya and Aeddula, 2022). Unrestrained HTN features an increased risk and rapid progression of CKD, while progressive renal disease can intensify uncontrolled HTN owing to volume expansion and elevated systemic vascular resistance (Segura and Ruilope, 2011). A physiological system highly significant for the regulation the blood pressure (BP) is the renin–angiotensin–aldosterone system (RAAS). The RAAS forms an essential hormonal and peptidergic endocrine system that regulates blood volume and systemic vascular resistance and determines damage to various target organs resulting from hypertension (Fountain and Lappin, 2022; Liu et al., 2020). The RAAS has autocrine-paracrine functions and is not limited to circulation but is also present locally in organ systems such as the kidneys, lungs, and the brain (Gelen et al., 2021). Both the systemic and local RAAS are independent; however, they are known to interact (Siragy and Carey, 2010). The RAAS elevates blood volume and arterial tone by enhancing sodium reabsorption, water reabsorption, and vascular tone and maintains blood volume and arteriolar tone on a long-term basis (Figure 1). A coordinated effect on the cardiovascular system and kidneys by RAAS controls the fluid and electrolyte balance of the body (Remuzzi et al., 2005). The RAAS is implicated in chronic alterations in blood pressure, while the baroreceptor reflex regulates minor and rapid shifts. Due to RAAS being crucial for the maintenance of BP and electrolyte balance, an inapposite activation can lead to hypertension (Fountain and Lappin, 2022). Overactivity of the RAAS is known to be conducive to the pathogenesis of CKD mediated by intracapillary hypertension and elevated ultrafiltration of plasma proteins, along with the non-hemodynamic effect of angiotensin and augmentation of aldos, which contributes to renal and cardiac injury (Georgianos and Agarwal, 2018). All the components of the RAAS are present in the kidney, which are independent of the systemic RAAS. The angiotensin II (AT II) synthesized intra-renally controls the glomerular hemodynamics and the tubular sodium transport; however, it also activates various inflammatory and fibrotic pathways, subsequently increasing connective tissue production and extracellular matrix deposition (Mezzano et al., 2001). In diabetics, intrarenal RAAS activation is observed in early nephropathy, which forms the leading cause of CKD (Siragy and Carey, 2010). The significance of RAAS in modulating BP makes it a target of choice for pharmacological invention in the management of CKD; hence, multifocal inhibition of RAAS is the first-line antihypertensive therapy recommended by current guidelines and is provided as mono or dual therapy. The RAAS inhibitors have emerged as superior antihypertensive drugs in slowing the advancement of nephropathy to end-stage renal disease by reduction of proteinuria and optimized BP control; however, they are limited by the development of hyperkalemia in a large section of patients having proteinuria, leading to discontinued use or administration at suboptimal doses to prevent the development of hyperkalemia in such patients (Schaefer and Gales, 2016 16). Drugs that act on RAAS to effectuate renoprotection can 1) cause a direct inhibition of the synthesis and release of renin (renin inhibitors); 2) inhibit AII (ACE inhibitors); 3) lead to antagonization of the receptor effects of AII (ARBs); 4) antagonize the aldosterone-receptor, also called antimineralocorticoid (MCRA); and 5) simultaneously inhibit the neutral endopeptidase and angiotensin-converting enzyme (vasopeptidase). The RAAS modulators are significant drugs of choice to treat chronic diseases such as heart failure, myocardial infarction, hypertension, diabetes mellitus, and chronic kidney diseases; however, comparative studies that illustrate their different aspects are lacking. Such studies can help the healthcare provider prescribe the best choice of drug, as per the presentation of the patient. The RAAS inhibitors are currently the first line of drugs recommended to combat hypertension in CKD. They, however, present a wide spectrum of chemical structures with varying mechanisms of action and dissimilar treatment outcomes. In the current review, a detailed comprehensive head-to-head comparison of renin inhibitors, ACE inhibitors, and ARBs in CKD shall be drawn (Figure 2).

## 2 Compounds, structure, and binding

Aliskiren stands as the first direct renin inhibitor approved by FDA to date. It is a monomethoxy benzene and a monocarboxylic acid amide featuring a 3-methoxypropoxy group at the 2-position and a multi-substituted branched alkyl substituent at the 4-position (Aliskiren, 2022). Aliskiren is a transition-state mimetic that blocks the catalytic function of renin by forming a hydrogen bond between the central hydroxyl group and amino group to the catalytic Asp32 and Asp215 residues; however, no interaction with the S2 or S4 binding sites of renin is observed due to lack of large P4–P2 spacing backbone. A large hydrophobic S3–S1 super pocket accommodates the P3–P1 pharmacophore of aliskiren, while the S3sp sub-pocket accommodates the aromatic alkoxy sidechain (Wood et al., 2003) (Table 2).

**TABLE 2:** Molecular structural details of RAAS inhibitors (Ref: PubChem).

**Class**	**Drug**		**Formula and Molecular weight**	**IUPAC name**	**International Chemical Identifier**	**Net charge**	**2D Structure**
**Renin Inhibitor**	Aliskiren		C30H53N3O6, 551.8g/mol	(2S,4S,5S,7S)-5-amino-N-(3-amino-2,2-dimethyl-3-oxopropyl)-4-hydroxy-7-[[4-methoxy-3-(3-methoxypropoxy)phenyl]methyl]-8-methyl-2-propan-2-ylnonanamide	1S/C30H53N3O6/c1-19(2)22(14-21-10-11-26(38-8)27(15-21)39-13-9-12-37-7)16-24(31)25(34)17-23(20(3)4)28(35)33-18-30(5,6)29(32)36/h10-11,15,19-20,22-25,34H,9,12-14,16-18,31H2,1-8H3,(H2,32,36)(H,33,35)/t22-,23-,24-,25-/m0/s1	0	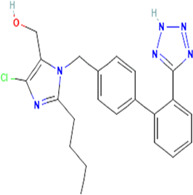
**ACE inhibitors**	Functional group-Sulfhydryl	captopril	C9H15NO3S, 217.29g/mol	(2*S*)-1-[(2*S*)-2-methyl-3-sulfanylpropanoyl]pyrrolidine-2-carboxylic acid	1S/C9H15NO3S/c1-6(5-14)8(11)10-4-2-3-7(10)9(12)13/h6-7,14H,2-5H2,1H3,(H,12,13)/t6-,7+/m1/s1	0	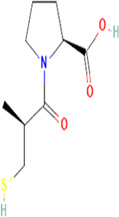
		Alacepril	C20H26N2O5S, 406.5g/mol	(2S)-2-[[(2S)-1-[(2S)-3-acetylsulfanyl-2-methylpropanoyl]pyrrolidine-2-carbonyl]amino]-3-phenylpropanoic acid	1S/C20H26N2O5S/c1-13(12-28-14(2)23)19(25)22-10-6-9-17(22)18(24)21-16(20(26)27)11-15-7-4-3-5-8-15/h3-5,7-8,13,16-17H,6,9-12H2,1-2H3,(H,21,24)(H,26,27)/t13-,16+,17+/m1/s1	0	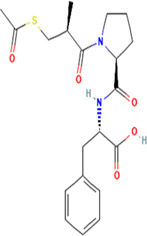
		zofenopril	C22H23NO4S, 429.6 g/mol	(2*S*,4*S*)-1-[(2*S*)-3-benzoylsulfanyl-2-methylpropanoyl]-4-phenylsulfanylpyrrolidine-2-carboxylic acid	1S/C22H23NO4S2/c1-15(14-28-22(27)16-8-4-2-5-9-16)20(24)23-13-18(12-19(23)21(25)26)29-17-10-6-3-7-11-17/h2-11,15,18-19H,12-14H2,1H3,(H,25,26)/t15-,18+,19+/m1/s1	0	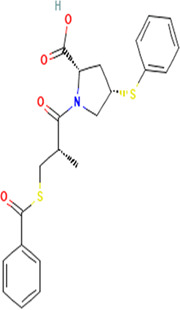
	Functional group-Dicarboxylate	**Enalapril**	C20H28N2O5, 376.4 g/mol	(2*S*)-1-[(2*S*)-2-[[(2*S*)-1-ethoxy-1-oxo-4-phenylbutan-2-yl] amino]propanoyl]pyrrolidine-2-carboxylic acid	1S/C20H28N2O5/c1-3-27-20(26)16(12-11-15-8-5-4-6-9-15)21-14(2)18(23)22-13-7-10-17(22)19(24)25/h4-6,8-9,14,16-17,21H,3,7,10-13H2,1-2H3,(H,24,25)/t14-,16-,17-/m0/s1	0	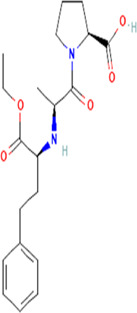
		**Ramipril**	C23H32N2O5 416.5 g/mol	(2*S*,3*aS*,6*aS*)-1-[(2*S*)-2-[[(2*S*)-1-ethoxy-1-oxo-4-phenylbutan-2-yl]amino]propanoyl]-3,3*a*,4,5,6,6*a*-hexahydro-2*H*-cyclopenta[b]pyrrole-2-carboxylic acid	1S/C23H32N2O5/c1-3-30-23(29)18(13-12-16-8-5-4-6-9-16)24-15(2)21(26)25-19-11-7-10-17(19)14-20(25)22(27)28/h4-6,8-9,15,17-20,24H,3,7,10-14H2,1-2H3,(H,27,28)/t15-,17-,18-,19-,20-/m0/s1	0	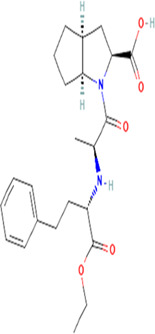
		**Quinapril**	C25H30N2O, 438.5 g/mol	(3*S*)-2-[(2*S*)-2-[[(2*S*)-1-ethoxy-1-oxo-4-phenylbutan-2-yl]amino]propanoyl]-3,4-dihydro-1*H*-isoquinoline-3-carboxylic acid	1S/C25H30N2O5/c1-3-32-25(31)21(14-13-18-9-5-4-6-10-18)26-17(2)23(28)27-16-20-12-8-7-11-19(20)15-22(27)24(29)30/h4-12,17,21-22,26H,3,13-16H2,1-2H3,(H,29,30)/t17-,21-,22-/m0/s1	0	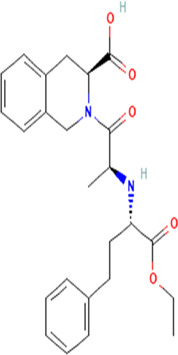
		**Perindopril**	C19H32N2O,368.5 g/mol	(2*S*,3*aS*,7*aS*)-1-[(2*S*)-2-[[(2*S*)-1-ethoxy-1-oxopentan-2-yl]amino]propanoyl]-2,3,3*a*,4,5,6,7,7*a*-octahydroindole-2-carboxylic acid	1S/C19H32N2O5/c1-4-8-14(19(25)26-5-2)20-12(3)17(22)21-15-10-7-6-9-13(15)11-16(21)18(23)24/h12-16,20H,4-11H2,1-3H3,(H,23,24)/t12-,13-,14-,15-,16-/m0/s1	0	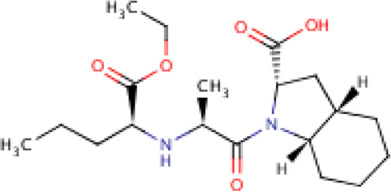
		**Lisinopril**	C21H31N3O5, 405.5g/mol	(2*S*)-1-[(2*S*)-6-amino-2-[[(1*S*)-1-carboxy-3-phenylpropyl]amino]hexanoyl]pyrrolidine-2-carboxylic acid	1S/C21H31N3O5/c22-13-5-4-9-16(19(25)24-14-6-10-18(24)21(28)29)23-17(20(26)27)12-11-15-7-2-1-3-8-15/h1-3,7-8,16-18,23H,4-6,9-14,22H2,(H,26,27)(H,28,29)/t16-,17-,18-/m0/s1	0	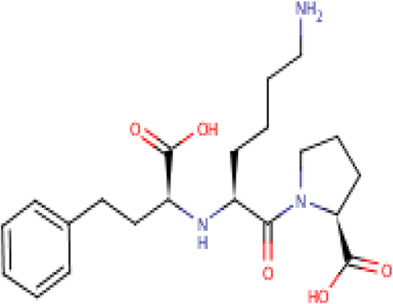
		**Benazepril**	C24H28N2O5, 424.5 g/mol	2-[(3S)-3-[[(2S)-1-ethoxy-1-oxo-4-phenylbutan-2-yl]amino]-2-oxo-4,5-dihydro-3H-1-benzazepin-1-yl]acetic acid	1S/C24H28N2O5/c1-2-31-24(30)20(14-12-17-8-4-3-5-9-17)25-19-15-13-18-10-6-7-11-21(18)26(23(19)29)16-22(27)28/h3-11,19-20,25H,2,12-16H2,1H3,(H,27,28)/t19-,20-/m0/s1	0	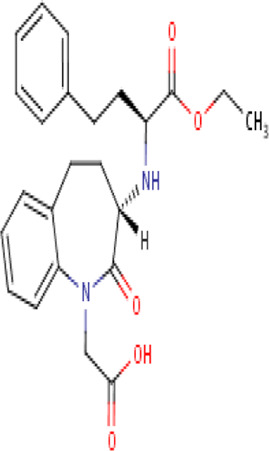
		**Imidapril**	C20H27N3O6 405.4 g/mol	(4*S*)-3-[(2*S*)-2-[[(2*S*)-1-ethoxy-1-oxo-4-phenylbutan-2-yl]amino]propanoyl]-1-methyl-2-oxoimidazolidine-4-carboxylic acid	1S/C20H27N3O6/c1-4-29-19(27)15(11-10-14-8-6-5-7-9-14)21-13(2)17(24)23-16(18(25)26)12-22(3)20(23)28/h5-9,13,15-16,21H,4,10-12H2,1-3H3,(H,25,26)/t13-,15-,16-/m0/s1	0	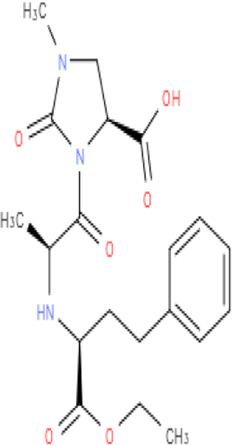
		**Trandolapril**	C24H34N2O5, 430.5 g/mol	(2*S*,3*aR*,7*aS*)-1-[(2*S*)-2-[[(2*S*)-1-ethoxy-1-oxo-4-phenylbutan-2-yl]amino]propanoyl]-2,3,3*a*,4,5,6,7,7*a*-octahydroindole-2-carboxylic acid	1S/C24H34N2O5/c1-3-31-24(30)19(14-13-17-9-5-4-6-10-17)25-16(2)22(27)26-20-12-8-7-11-18(20)15-21(26)23(28)29/h4-6,9-10,16,18-21,25H,3,7-8,11-15H2,1-2H3,(H,28,29)/t16-,18+,19-,20-,21-/m0/s1	0	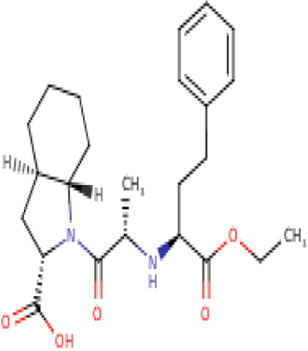
		**Cilazapril**	C22H31N3O, 417.5 g/mol	(4*S*,7*S*)-7-[[(2*S*)-1-ethoxy-1-oxo-4-phenylbutan-2-yl]amino]-6-oxo-1,2,3,4,7,8,9,10-octahydropyridazino[1,2-a]diazepine-4-carboxylic acid	1S/C22H31N3O5/c1-2-30-22(29)18(13-12-16-8-4-3-5-9-16)23-17-10-6-14-24-15-7-11-19(21(27)28)25(24)20(17)26/h3-5,8-9,17-19,23H,2,6-7,10-15H2,1H3,(H,27,28)/t17-,18-,19-/m0/s1	0	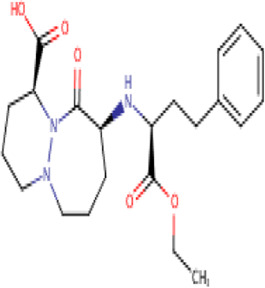
	Functional group-Phosphonate	**Fosinopril**	C30H46NO7,563.7 g/mol	(2*S*,4*S*)-4-cyclohexyl-1-[2-[(2-methyl-1-propanoyloxypropoxy)-(4-phenylbutyl)phosphoryl]acetyl]pyrrolidine-2-carboxylic acid	1S/C30H46NO7P/c1-4-28(33)37-30(22(2)3)38-39(36,18-12-11-15-23-13-7-5-8-14-23)21-27(32)31-20-25(19-26(31)29(34)35)24-16-9-6-10-17-24/h5,7-8,13-14,22,24-26,30H,4,6,9-12,15-21H2,1-3H3,(H,34,35)/t25-,26+,30?,39?/m1/s1	0	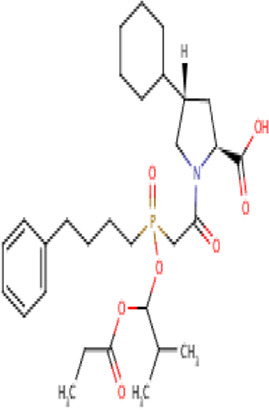
**ARBs**	Losartan		C22H23ClN6O, 422.9 g/mol	[2-butyl-5-chloro-3-[[4-[2-(2*H*-tetrazol-5-yl)phenyl]phenyl]methyl]imidazol-4-yl]methanol	1S/C22H23ClN6O/c1-2-3-8-20-24-21(23)19(14-30)29(20)13-15-9-11-16(12-10-15)17-6-4-5-7-18(17)22-25-27-28-26-22/h4-7,9-12,30H,2-3,8,13-14H2,1H3,(H,25,26,27,28)	0	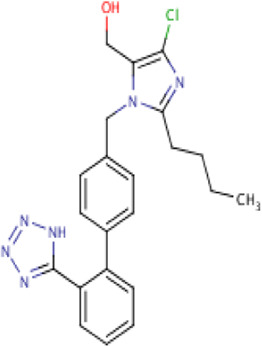
	Irbesartan		C25H28N6O, 428.5 g/mol	2-butyl-3-[[4-[2-(2*H*-tetrazol-5-yl)phenyl]phenyl]methyl]-1,3-diazaspiro[4.4]non-1-en-4-one	1S/C25H28N6O/c1-2-3-10-22-26-25(15-6-7-16-25)24(32)31(22)17-18-11-13-19(14-12-18)20-8-4-5-9-21(20)23-27-29-30-28-23/h4-5,8-9,11-14H,2-3,6-7,10,15-17H2,1H3,(H,27,28,29,30)	0	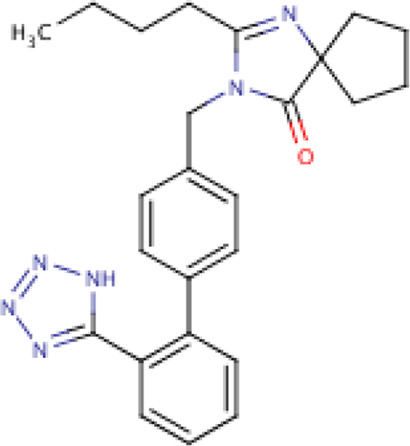
	Olmesartan		C24H26N6O3,446.5 g/mol	5-(2-hydroxypropan-2-yl)-2-propyl-3-[[4-[2-(2*H*-tetrazol-5-yl)phenyl]phenyl]methyl]imidazole-4-carboxylic acid	1S/C24H26N6O3/c1-4-7-19-25-21(24(2,3)33)20(23(31)32)30(19)14-15-10-12-16(13-11-15)17-8-5-6-9-18(17)22-26-28-29-27-22/h5-6,8-13,33H,4,7,14H2,1-3H3,(H,31,32)(H,26,27,28,29)	0	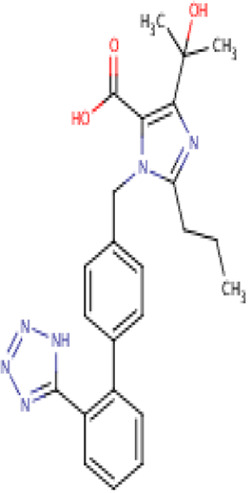
	candesartan		C24H20N6O3, 440.5 g/mol	2-ethoxy-3-[[4-[2-(2*H*-tetrazol-5-yl)phenyl]phenyl]methyl]benzimidazole-4-carboxylic acid	1S/C24H20N6O3/c1-2-33-24-25-20-9-5-8-19(23(31)32)21(20)30(24)14-15-10-12-16(13-11-15)17-6-3-4-7-18(17)22-26-28-29-27-22/h3-13H,2,14H2,1H3,(H,31,32)(H,26,27,28,29)	0	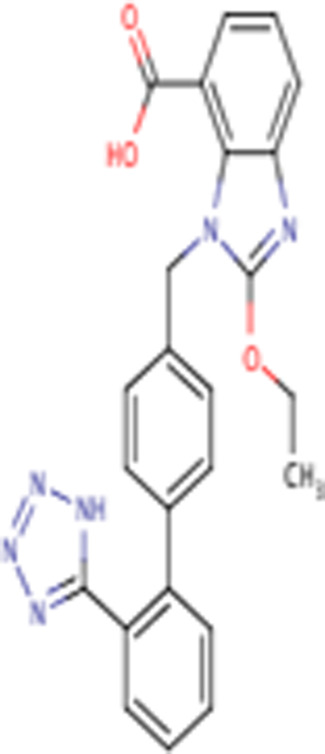
	valsartan		C24H29N5O3 , 435.5 g/mol	(2*S*)-3-methyl-2-[pentanoyl-[[4-[2-(2*H*-tetrazol-5-yl)phenyl]phenyl]methyl]amino]butanoic acid	1S/C24H29N5O3/c1-4-5-10-21(30)29(22(16(2)3)24(31)32)15-17-11-13-18(14-12-17)19-8-6-7-9-20(19)23-25-27-28-26-23/h6-9,11-14,16,22H,4-5,10,15H2,1-3H3,(H,31,32)(H,25,26,27,28)/t22-/m0/s1	0	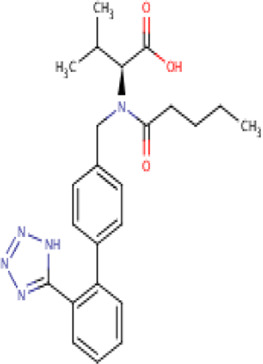
	**Fimasartan**		C27H31N7O,S 501.6 g/mol	2-[2-butyl-4-methyl-6-oxo-1-[[4-[2-(2*H*-tetrazol-5-yl)phenyl]phenyl]methyl]pyrimidin-5-yl]-*N*,*N*-dimethylethanethioamide	1S/C27H31N7OS/c1-5-6-11-24-28-18(2)23(16-25(36)33(3)4)27(35)34(24)17-19-12-14-20(15-13-19)21-9-7-8-10-22(21)26-29-31-32-30-26/h7-10,12-15H,5-6,11,16-17H2,1-4H3,(H,29,30,31,32)	0	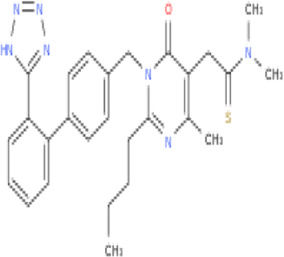
	**Azilsartan**		C25H20N4O5, 456.4 g/mol	2-ethoxy-3-[[4-[2-(5-oxo-4*H*-1,2,4-oxadiazol-3-yl)phenyl]phenyl]methyl]benzimidazole-4-carboxylic acid	1S/C25H20N4O5/c1-2-33-24-26-20-9-5-8-19(23(30)31)21(20)29(24)14-15-10-12-16(13-11-15)17-6-3-4-7-18(17)22-27-25(32)34-28-22/h3-13H,2,14H2,1H3,(H,30,31)(H,27,28,32)	0	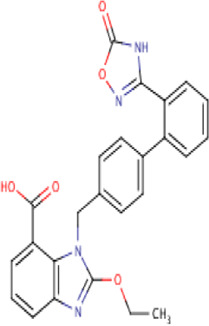
	**Telmisartan**		C33H30N4O2, 514.6 g/mol	2-[4-[[4-methyl-6-(1-methylbenzimidazol-2-yl)-2-propylbenzimidazol-1-yl]methyl]phenyl]benzoic acid	1S/C33H30N4O2/c1-4-9-30-35-31-21(2)18-24(32-34-27-12-7-8-13-28(27)36(32)3)19-29(31)37(30)20-22-14-16-23(17-15-22)25-10-5-6-11-26(25)33(38)39/h5-8,10-19H,4,9,20H2,1-3H3,(H,38,39)	0	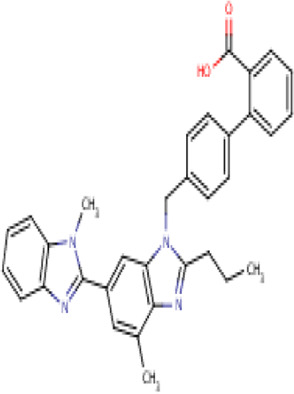
	**Eprosartan**		C23H24N2O4S, 424.5 g/mol	4-[[2-butyl-5-[(*E*)-2-carboxy-3-thiophen-2-ylprop-1-enyl]imidazol-1-yl]methyl]benzoic acid	1S/C23H24N2O4S/c1-2-3-6-21-24-14-19(12-18(23(28)29)13-20-5-4-11-30-20)25(21)15-16-7-9-17(10-8-16)22(26)27/h4-5,7-12,14H,2-3,6,13,15H2,1H3,(H,26,27)(H,28,29)/b18-12+	0	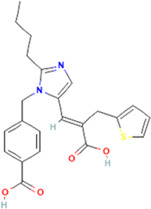

The basic structure of all ACE inhibitors is the same, but they differ in their functional groups, which can be carboxyl, sulfhydryl, or phosphinyl (Piepho, 2000). The prototype for ACE inhibitors is captopril, which is a pyrrolidinemonocarboxylic acid, an N-acylpyrrolidine, an alkanethiol, and an L-proline derivative, and has L-proline on nitrogen with a (2S)-2-methyl-3-sulfanylpropanoyl group. The binding of captopril to the active site of ACE occurs *via* the thiol moiety with the central Zn^2+^ ion of ACE (Caballero, 2020). The ACE–captopril complex gets stabilized by further hydrogen bonding with the carbonyl group of captopril through His353 and His513 residues (Bhuyan and Mugesh, 2011). Enalapril, lisinopril, benazepril, perindopril, quinapril, ramipril, trandolapril, moexipril, etc., are types of ACE inhibitors having different functional groups and were developed by transformations of captopril (Ulm et al., 1982; Caballero, 2020) (Table 2).

Seven types of ARBs are approved for clinical use worldwide. They have a common chemical structure of the biphenyl-tetrazole group and an imidazole group and show class effects. They are non-peptide Ang II receptor antagonists. Losartan serves as the prototype of ARBs, which is a biphenylyltetrazole having a 1,1′-biphenyl group attached to the 5-position with an extra imidazol-1-ylmethyl group trisubstituted at position 4. However, its binding affinity to **AT**_**1**_R is lower compared to the ARBs developed later (Miura et al., 2011). Only the tetrazole moiety of losartan forms a salt bridge with Arg167ECL2, while no polar interactions with other groups are reported. While the derived imidazole moiety of losartan may show polar interactions either through the hydrogen bond of methanol to Cys180ECL2 major chain or through nitrogen interaction with Tyr351.39, the distances and angles for hydrogen bonding are not optimal, which account for the lower binding affinity of losartan at **AT**_**1**_R (Zhang et al., 2015) (Table 2). However, docking simulation studies have speculated a common mode of binding for different ARBs. Zhang et al. (2015a), while studying the binding of olmesartan, reported that the drug was primarily anchored with the receptor by the residues Tyr-35^1.39^, Trp-84^2.60^, and Arg-167^ECL2^, which were similar to the antagonist ZD7155, confirming a common binding pattern of different ARBs.

## 3 Mechanism of action

Aliskiren is active in the RAAS and directly inhibits plasma renin activity, which forms the initial and limiting step in the RAAS (Sen et al., 2014). The juxtaglomerular cells occurring in the juxtaglomerular apparatus in the kidney secrete renin consistent with the variations in the blood volume and renal perfusion, as perceived by the macula densa in the distal tubule of the nephron. Renin converts angiotensinogen to angiotensin I, which is then converted to angiotensin II by the action of the angiotensin-converting enzyme present in the lung capillaries and endothelial cells in the kidneys. Angiotensin II binds to the AT_1_R, causing vasoconstriction, releases catecholamines, and enhances aldosterone secretion and sodium reabsorption (Jacobs et al., 2021). All such effects culminate in increasing blood pressure. Aliskiren inhibits renin, consequently blocking the change of angiotensinogen to angiotensin I and hence preventing the formation of angiotensin II. Aliskiren thereby reduces BP by lowering the amount of angiotensin II that reaches the AT_1_R (Pereira et al., 2019). Aliskiren is associated with reduced proteinuria in chronic proteinuric non-diabetic kidney disease patients.

ACE inhibitors block the conversion of ATI to ATII, stimulating the dilation of blood vessels (Herman et al., 2017). They inhibit the reuptake of norepinephrine and the release of catecholamines from the adrenal medulla (Marte et al., 2022). They cause a lowering of arteriolar resistance, an increase in venous capacity, lower resistance in blood vessels in the kidneys, and an increase in natriuresis (Sansoè et al., 2020). In experimental chronic renal failure, ACE inhibitors reduce glomerular capillary pressure and proteinuria and may cease the development of glomerular injury and loss of renal function (Opsahl et al., 1990).

ARBs effectuate a reduction in blood pressure by binding to the AT_1_R, which causes a decrease in aldosterone, vasopressin, and catecholamine release. In addition, they also lead to vascular vasodilation and inhibition of sodium and water reabsorption in the kidney.

## 4 Molecular basis of action

The accumulation of aliskiren in the plasma membrane is crucial for the effective inhibition of renin by the drug. Aliskiren has high lipophilicity and accumulates efficiently in the lipid bilayers. However, the cholesterol-rich domain formation in the direct vicinity of a (pro)-renin receptor can cause the membrane-accumulated aliskiren molecules to get expelled from the lipid bilayer, leading to the binding of the inhibitors to the active site from the extracellular fluid. This mechanism is particularly significant for the intracellular renin–angiotensin system (Sadeghpour et al., 2015).

Captopril, which is studied as the prototype of ACE inhibitors, is generally present as an equilibrium mixture of cis and trans isomers, with respect to the proline amide bond at neutral pH in the solution, and the angiotensin I-converting enzyme takes only the trans-state of the inhibitor which bears reciprocity with its substrate binding groove both architecturally and stereoelectronically (Tzakos et al., 2006).

Studies with losartan (the prototype for ARBs) report two main conformations in solution that position the tetrazole and imidazole moieties either in an anti or a syn orientation respective to the A phenyl ring plane. Losartan is observed to have a spontaneous insertion in the lipidic core in micellar and lipid bilayers, having embedded the AT_1_R. The drug moves from the aqueous environment to the lipid surface within the first few nanoseconds, expediating into the bilayer and percolating between the polar headgroups and the upper part of the alkyl chains of the lipids, and forming hydrogen bonds with the lipid glycerol backbone, phosphate groups, and isolated water molecules penetrating the lipid bilayer (Zervou et al., 2014).

## 5 Guideline recommendations for DRI, ACEIs, and ARBs in CKD

The recommendations are provided by the world bodies for the pharmacotherapy and management of hypertension in CKD. These recommendations aim to improve clinical practices and treatment outcomes, are drafted with inputs from field experts and other stakeholders, and are updated regularly to fill the gaps between targets and outcomes. The recommendations are not rigid, and the healthcare provider can consider resources and facilities available in addition to the nature and presentation of the patient for treatment; however, the recommendations aim to facilitate the management of hypertension in CKD. Numerous trials have inferred that RAAS inhibitors reduce the advancement of kidney disease in people presenting with BP and/or diabetes. RAAS inhibitors were observed to have considerably reduced kidney disease progression in comparison to placebo and other antihypertensive agents. There is no significant difference between ACEIs and ARBs when overall mortality, progression to ESRD, or their anti-proteinuric effects are concerned, and initially, the drug is selected based on patient preference, potential side effects, and cost. Various bodies provide guidelines for the management of CKD, and among them, the Kidney Disease Improving Global Outcomes (KDIGO) and the National Institute for Health and Care Excellence (NICE) are globally recognized bodies for the management of renal disorders, suggesting target BP in CKD (KDIGO 5). KDIGO guidelines suggest attaining a target of systolic blood pressure of <120 mm Hg, if tolerated, in adults with increased BP and CKD by using the standardized office BP measurement. This recommendation of a target BP of <120 mm Hg does not hold good when measured in a non-standardized manner. NICE recommends BP of below 140 mmHg for patients with CKD and an albumin creatinine ratio (ACR) under 70 mg/mmol and below 130 mmHg for patients with CKD and an ACR under 70 mg/mmol or above. RAAS inhibitors are recommended to obtain the target BP. When seen comparatively, clear and vivid recommendations are seen for ACEIs and ARBs but are lacking for DRI. Here, we provide the latest recommendations for the use of RAAS inhibitors for the management of BP in CKD as per KDIGO and NICE (Table 3).

**TABLE 3:** Recommendations for the use of RAAS inhibitors for management of BP in CKD as per KDIGO and NICE.

Diabetes	Recommendations (KDIGO and NICE)					
	DRI	ACEIs	ARBs	Albuminuria	Applicable for CKD patients that receive dialysis or a kidney transplant	Strength of recommendation
No	Not provided	Start	Start	Severely increased (G1–G4 and A3)	No	Strong
No	Not provided	Start	Start	Modestly elevated albuminuria (G1–G4 and A2)	No	Weak
Yes	Not provided	Start	Start	Modestly to severely elevated albuminuria (G1–G4, A2, and A3	No	Strong
No	—	—	Start	—	Kidney transplant	Strong

KDIGO recommends avoiding any combination of ACEI, ARB, and DRI therapy in diabetic and non-diabetic CKD patients. NICE recommends not offering RAAS inhibitors to CKD patients with a pretreatment serum K^+^ level of >5.0 mmol/L and discontinuing them when serum K^+^ reaches ≥6.0 mmol/L in patients.

## 6 Dosages, contraindications, and dosage modifications in renal impairments

It is mandatory, as per the current guidelines, to use RAAS inhibitors in patients with proteinuric CKD as first-line renoprotection since randomized trials have strongly demonstrated RAAS inhibitors to be superior in lowering the progression of nephropathy to end-stage renal disease when compared to other antihypertensive drug classes (Lv et al., 2013; Gant et al., 2022). They reduce urinary excretion of albumin, causing a delay in renal function deterioration, in addition to reducing hypertension, thus offering renoprotection (Alexandrou et al., 2019). Still, there is a lack of sufficient real-life data describing the actual use and dosage of RAAS inhibitors in high-risk patients. A supramaximal dose of a single inhibitor or combined use may provide increased renoprotection; however, their maximal and/or combined use is impeded by the development of hyperkalemia, indicating a limited opportunity to block RAAS (Gant et al., 2022). The DRI (aliskiren) was hypothesized to have a more effective anti-proteinuric activity as it does not induce the renin escape phenomenon as seen with ACEIs and ARBs. However, aliskiren was associated with worsened renal and cardio outcomes in various studies and was consequently prohibited in diabetics and in combination therapy. The dosage is generally 150 mg PO once daily, which can be increased to 300 mg PO once daily in case the blood pressure is not adequately controlled (Simeoni et al., 2019). ACEIs and ARBs cause efferent arteriolar dilation in CKD patients, leading to a sharp decline in GFR (>15% from baseline), associated with a proportionate increase in the serum creatinine level within the first week of starting the therapy. This is more often seen in patients presenting with congestive heart failure, patients who are on diuretic or non-steroidal anti-inflammatory drug therapy, and patients prescribed high doses of ACE inhibitors or ARBs. In most patients, the combination of ACE inhibitors and ARBs can be continued safely until the increase in serum creatinine is less than 30% and can be discontinued if the serum creatinine level increases more than 30% or if the potassium level in serum is 5.6 mmol per L or higher (Munar et al., 2007). However, careful titration of dosages followed by weekly monitoring of renal function and potassium levels is necessary until the values return to baseline, and contraindicated drugs should be considered rather than denying ACEI and ARB therapy to patients. A comparative list of dosages of contraindicated drugs and dosage modifications is provided in Table 4.

**TABLE 4:** Agents for renin–angiotensin–aldosterone blockade, their doses, contraindicated drugs, and dosage modification in CKD.

Class	Drug	Usual dose, range (mg/d)*	Contraindicated	Dose adjustments based on GFR (mL/min/1.73 m^2^) (percentage of usual dosage)			Remarks for prescription in CKD
				30–59	10–29	<10	
	Benazepril	10–40 mg/day (divided q12–24h)	Aliskiren sacubitril/valsartan	75%	50%	25%	An increased risk of hyperkalemia is associated with CKD patients. Not recommended in combination with ARBs or direct renin inhibitor. Avoid during pregnancy.
	Captopril	25–50 mg q8–12h	Aliskiren sacubitril/valsartan	75%	50%–75%	50%	
	Enalapril	5–40 mg/day (divided q12–24h)	Aliskiren protein a column sacubitril/valsartan	50%–100%	50%	25%	
	Fosinopril	10–40 mg/day (divided q12–24h)	Aliskiren protein a column sacubitril/valsartan	—	—	75%–100%	
	Lisinopril	10–40 mg q24h	Aliskiren protein a column sacubitril/valsartan	50%–75%	50%	25%–50%	
	Moexipril	7.5–30 mg/day (divided q12–24h)	Aliskiren protein a column sacubitril/valsartan	50%	50%	50%	
	Perindopril	4–16 mg q24h	Aliskiren protein a column sacubitril/valsartan	50%	Max dose of 2 mg q48h	Max dose of 2 mg q48h	
	Quinapril	10–80 mg/day (divided q12–24h)	Aliskiren protein a column sacubitril/valsartan	50%	25%–50%	25%	
	Ramipril	2.5–20 mg/day (divided q12–24h)	Aliskiren protein a column sacubitril/valsartan	50%	25%–50%	25%	
	Trandolapril	1–4 mg/day (divided q12–24h)	Aliskiren protein a column sacubitril/valsartan	—	50%	50%	
ARBs	Azilsartan	40–80	Aliskiren	—	—	—	CKD patients are at an increased risk of hyperkalemia. Not recommended in patients with a history of angioedema with ARBs. Avoid during pregnancy. Not recommended in combination with ACE inhibitors or direct renin inhibitor.
	Candesartan	16–32 mg/day (divided q12–24h)	Aliskiren	—	—	—	
	Eprosartan	600–800	Aliskiren	—	—	—	
	Irbesartan	150–300 mg q24h	Aliskiren	—	—	—	
	Losartan	50–100 mg q24h	Aliskiren	—	—	—	
	Olmesartan	20–40 mg q24h	Aliskiren	—	—	50%	
	Telmisartan	40–80 mg q24h	Aliskiren elagolix	—	—	—	
	Valsartan	80–320 mg q24h	Aliskiren	—	—	—	
Direct renin inhibitor	Aliskiren	150–300	Azilsartan, benazepril, candesartan, captopril, enalapril, eprosartan, fosinopril, irbesartan, lisinopril, losartan, moexipril, olmesartan, perindopril, quinapril, ramipril, sacubitril/valsartan, telmisartan, trandolapril, and valsartan	—	—	—	Not recommended in combination with ACE inhibitors or ARBs. A higher risk of hyperkalemia in CKD patients. Avoid during pregnancy.

Source: https://reference.medscape.com/; https://www.med.umich.edu/1info/FHP/practiceguides/kidney/CKD.pdf.

## 7 Pharmacokinetics

CKD is a condition commonly connected with changes in the pharmacokinetics of the dispensed drugs, consequently leading to drug dose adjustments. The condition has varying physiological effects. The drug clearance decreases, but the volume of distribution may not vary or may increase. In addition, there is an altered elimination of drugs by the renal and non-renal routes (Rowland Yeo et al., 2011; Lea-Henry et al., 2018). The pharmacokinetics of RAAS inhibitors is also altered in CKD to varying effects. Aliskiren, being a transition-state mimetic, has high aqueous solubility and high hydrophilicity favoring oral bioavailability (Allikmets, 2007). It shows high potency in inhibiting RAAS, and 50% of renin inhibition occurs at a concentration of 0.6 nmol/L (Vaidyanathan et al., 2008a). Post oral administration, about 5% of aliskiren is absorbed rapidly, showing a peak plasma time of about 1–3 h (Waldmeier et al., 2007). It has poor bioavailability (2%–6%), which is compensated by its high solubility and inhibitory effect. Aliskiren moderately binds to plasma proteins, 47%–51% being independent of the concentration. It undergoes a minimal hepatic metabolism *via* CYP3A4 and neither inhibits nor induces the cytochrome P450 system (Rashid, 2008). Preclinical studies report P-gp to be the major efflux system for intestinal absorption and elimination *via* biliary excretion. The elimination of aliskiren is primarily an unchanged drug through the hepatobiliary route, which accounts for about 80% of the drug in the plasma post oral administration. The major excretion of aliskiren occurs *via* the biliary/fecal route; however, the urine yields 0.6% of the dose. After 7–8 days of once-daily dosing, the steady-state plasma concentrations are reached, with an accumulation factor of approximately 2, while the optimum effect is achieved within 2 weeks (Vaidyanathan et al., 2008b; Vaidyanathan et al., 2008a). Once the peak is reached, the plasma concentrations of aliskiren lower in a multiphasic manner (Novo et al., 2009). No reports confirm gender or race to affect the pharmacokinetics of aliskiren. While being co-administered with a wide range of potential concomitant medications, no significant increases in exposure were observed, except with P-glycoprotein inhibitors. When taken with a high-fat meal, a decrease in the mean AUC and Cmax of aliskiren by 71% and 85%, respectively, was observed (Tabassum, 2011). Advanced stages of CKD are reported to moderately alter the pharmacokinetics of aliskiren. It is minimally dialyzable, and the dosage adjustment of aliskiren in CKD is not based on pharmacokinetic considerations. In Japanese patients presenting with hypertension and renal dysfunction, a dose-dependent increase was reported in trough plasma aliskiren levels that reached a steady state after 2–4 weeks of aliskiren treatment or dose increment (Ito et al., 2010). Vaidyanathan et al. (2008b) reported that a single dose of 300 mg aliskiren caused an increase in exposure to mild, moderate, and severe renal impairment, with AUCs being 157%, 290%, and 181% higher, respectively, when compared to healthy subjects (Vaidyanathan et al., 2008a). Khadzhynov et al. (2012) also reported a marginally higher exposure because of aliskiren in ESRD patients. In ESRD patients who received one dose of 300 mg aliskiren, the area under the AUC from time zero to infinity was elevated by 61% and 41% for hemodialysis at 48 h and 1 h, respectively, when compared with the healthy subjects. In normal subjects, the peak plasma drug concentration was 17% higher and 16% lower following hemodialysis at 48 h and 1 h, respectively, suggesting that aliskiren causes a moderately higher exposure in ESRD patients (Khadzhynov et al., 2012).

The pharmacokinetics of ACEIs is difficult to assess and are poorly characterized in terms of their quantitative pharmacokinetics. This is owing to numerous factors complicating their analysis, especially their physicochemical differences and differences in binding affinity, potency, lipophilicity, and depot effect (Dzau et al., 2001; Levitt and Schoemaker, 2006). The ACEIs have a common pathway of reducing BP but vary in absorption, half-life, protein binding, and metabolic disposition. All of them except lisinopril and captopril are administered as prodrugs, which are changed by hepatic esterolysis into active diacid metabolites. Captopril and lisinopril are adequately bioavailable when given orally and are hence prescribed as active drugs (Hoyer et al., 1993). The concentration of the free inhibitor in tissue is influenced by pharmacologic factors such as frequency and amount of dose, plasma half-life, bioavailability, tissue penetration, and finally, the volume that can be retained at the tissue level. For ACEIs, their bioavailability and half-life in the blood form important factors in the selection of an ACEI dose. Most of the ACEIs are generally cleared renally, involving filtration and a varying level of secretion by the organic anion secretory pathway. However, considerable hepatic and renal elimination is observed for fosinoprilat and trandolaprilat (Sica et al., 1991). Since renal elimination is the major elimination pathway of ACEIs, renal insufficiency leads to reduced elimination of most ACEIs, causing altered pharmacokinetic properties (Hoyer et al., 1993). In a physiology-based PK model for ACEIs, Ramusovic and Laeer (2012) prognosticated a sharp Ang I increase and Ang II decrease as a consequence of ACE inhibition in impaired renal condition than in normal renal function for benazepril, predicting altered pharmacokinetics (Ramusovic and Laeer, 2012). Earlier studies have reported altered pharmacokinetics for captopril in CKD as a significant reduction in drug clearance (CL) was observed in patients with varying disease severity (Duchin et al., 1984; Giudicelli et al., 1984). Model-based studies by Rasool et al. (2021) have also predicted changes in captopril, Cmax, AUC, and clearance in CKD populations with varying levels of severity and postulated PK parameters to be between a twofold range (Rasool et al., 2021). In the case of benazepril and cilazapril, Versypt et al. (2017), in their model-based studies, reported drug diacid concentration to accumulate more quickly and largely in impaired renal function in comparison to normal renal function, owing to impaired renal clearance mechanisms (Versypt et al., 2017). Also, the lowering of Ang II concentrations was attained by increased doses of the same drug as anticipated for normal renal function. In patients requiring hemodialysis, there is a considerable variation in the efficacy of clearance for prodrugs or active drugs and their metabolites by either hemodialysis or peritoneal dialysis. Captopril and enalapril are effectively eliminated by hemodialysis and require a supplemental dose after dialysis, while poor elimination of quinapril or cilazapril by peritoneal dialysis or hemodialysis is reported. The pharmacokinetic alteration of the ACEIs requires dosage adjustments in chronic renal failure for the ACEIs administered; the required dosage is 25%–50% of the dose prescribed for patients with normal renal function (Hoyer et al., 1993).

The pharmacokinetic profile of ARBs varies as per the difference in their molecular structure that causes a differential binding affinity to the receptor. A variation in solubility in lipids, absorption and distribution, biotransformation, bioavailability, plasma half-life, protein binding, and systemic elimination consequently influences the onset time, period of action, and efficacy of the ARBs. There is a rapid absorption of ARBs post oral administration (peak plasma levels achieved in 0.5–4 h) and an extended bioavailability (13% for eprosartan to 60%–80% for irbesartan). Most of the ARBs show high plasma protein binding (95%–100%) and a variable plasma elimination half-life varying from short to intermediate to long (1–4 h for candesartan and losartan, 5–10 h for eprosartan and valsartan, and 11–38 h for telmisartan, candesartan, and irbesartan). There is no significant accumulation of drugs and their active metabolites after repeated dosing, except for telmisartan (100%). Also, a major portion of the orally administered dose of ARBs undergoes biliary and fecal elimination; however, telmisartan (2%) and candesartan (33%) are excreted *via* urine. Data describing the pharmacokinetics of ARBs in CKD are insufficient. However, there are variable changes in PK reported in impaired renal function over the spectrum of the ARBs. In mild and intermediate renal disorders, the pharmacokinetic changes do not imply a change in the dose (Israili, 2000). Losartan undergoes renal and biliary excretion and is not dialyzed. Across the range of renal insufficiency, the renal clearance for losartan was reported to have decreased from 50 ± 19 mL/min in the normal group to 2.3 ± 0.9 mL/min in the severe group; however, the area under the plasma concentration curve remained unchanged (Sica et al., 1995; Burnier, 2001). In hemodialysis patients also, the pharmacokinetics of losartan did not alter significantly; hence, no adjustments in dose were required. Also, post-dialysis supplementation is not required for losartan since there is negligible dialyzability of losartan (Sica et al., 2000). Telmisartan presents an extended duration of action, with 24 h mean terminal half-life (being the longest in its group) and excessive tissue penetration, and shows a high affinity for angiotensin receptors. Telmisartan has only 1% renal excretion; hence, it is most unlikely to be affected by renal impairments (Bichu et al., 2009). Patients presenting renal insufficiency have a slowed-down renal clearance of eprosartan; however, with a minimal fraction of eprosartan being cleared by the kidney, dose adjustment is not necessary for patients with chronic renal failure (Bottorf and Tenero, 1999). The pharmacokinetics of ARBs is noted to vary the least in renal impairments and hence puts forward a favorable option in patients with CKD, with less need for dose adjustments.

## 8 Treatment outcomes

There is no therapeutic agent specifically for CKD, but control of blood pressure is crucial in the treatment of CKD (NHS, 2022). The RAAS inhibitors are the agents frequently used to attain this goal; in addition, they lower proteinuria in diabetic and non-diabetic patients, independent of BP reduction (Lewis et al., 1993; Brenner et al., 2001; Sarafidis et al., 2007). CKD randomized controlled trials conducted in adults have demonstrated slow progression of CKD with RAAS inhibitors and are principally recognized as first-line management (ACEIs and ARBs) of hypertension in CKD patients with proteinuria (Pugh et al., 2019; Gaudreault-Tremblay and Foster, 2020). Even though they inhibit the RAAS, the mechanisms are distinct and hence have varying efficacies and need to be prescribed with the evaluation of the patient presentation. Head-to-head comparison of the treatment outcomes can be convoluted but needs to be addressed for improving the precision of choice for treatment options (Table 5).

**TABLE 5:** Advantages and disadvantages of DRI, ACEIs, and ARBs in CKD.

	Advantages	Disadvantages
DRI	• Decreases systolic and diastolic BP.	• May be associated with symptomatic hypotension
	• Causes a progressive decrease in urinary albumin-to-creatinine ratio.	• Associated with hyperkalemia
	• May reduce sympathetic hyperactivity in patients with CKD.	• Monotherapy of aliskiren in CKD is not commonly practiced and is less investigated, yielding inconclusive results.
	• Inhibits brain natriuretic peptide, high-sensitivity C-reactive protein, and diacron-reactive oxygen metabolite.	• Combination treatments are more favored
	• Decreases urinary albumin/creatinine ratios in IgA nephropathy patients.	• Has no additional benefit for renoprotection or increase in adverse events in non-diabetic CKD patients, except for more hyperkalemia events
	• Mitigates oxidative stress and may improve the functional status of tubules.	• Is expensive
ACEIs	• Reduce the systemic vascular resistance, thereby decreasing hypertension.	• Lead to a compensatory rise in renin levels due to loss of negative feedback inhibition of renin
	• Reduce the incidence of progression to overt proteinuria.	• Have minimal effect on local Ang II production
	• Reduce the rate of GFR decline to levels similar to those associated with normal aging.	• Have been associated with instances of acute liver injury, acute kidney injury, and acute renal failure.
	• Significantly slow the rate of decline in creatinine clearance.	• Can cause an idiosyncratic reaction of ACEI-induced cough.
	• Reduce the markers of vascular microinflammation.	• In the diabetic population with renal transplants, ACEIs showed no association with improved clinical outcomes.
	• Increase the restoration of normoalbuminuria.	• ACEIs seem to have no benefit or no adversities at advanced stages
	• Are more effective in decelerating the progression to end-stage renal disease in non-diabetic patients presenting nephropathy when compared to other antihypertensives	• Discontinuation of ACEIs is common, especially in patients with lower eGFR.
ARBs	• Inhibit the vasoconstricting activity on smooth muscles, hence lowering blood pressure.	• Can raise the levels of renin, angiotensin I, and angiotensin II as a result of feedback inhibition.
	• The BP-lowering efficacy of ARBs is similar or numerically higher compared to ACE inhibitors when using ambulatory BP measurements. A numerically higher reduction in office systolic BP with ARBs is reported compared to ACE inhibitors.	• Can cause hypotension and/or renal failure in patients with heart failure presenting hypotension or bilateral renal artery stenosis
	• Although statistically insignificant, a better decrease in left ventricular mass index with ARBs is observed (13%) than with ACE inhibitors by 10%.	• ARBs are better tolerated than angiotensin-converting enzyme inhibitors
	• Have a lower rate of discontinuation	

Aliskiren is a relatively new candidate for RAAS inhibition and was deemed to overcome the elevated plasma renin concentration and activity associated with ACEI and ARB therapy (Angeli et al., 2014). The clinical treatment outcomes with aliskiren are still being evaluated and have revealed ambivalent results in CKD. Numerous studies have reported favorable results, while many have reported no beneficial treatment outcomes with aliskiren (Verdecchia et al., 2010; Bangalore et al., 2013; Chen et al., 2013; Zhang et al., 2015b; Catalá-López et al., 2016; Zheng et al., 2017). In 2020, Zhao et al., in their study, strongly suggested that aliskiren has BP lowering efficiency in essential hypertension but no significant outcome in the treatment of renal impairments (Zhao et al., 2020). Dhakarwal et al. (2014), in their study, acknowledged the lack of ample evidence for the renal benefit of aliskiren in diabetics beyond that offered by ACEIs or ARBs but suggested that aliskiren be used as a monotherapy, with careful monitoring, in the diabetic kidney, considering it an equivalent alternative to ACEIs and ARBs owing to its antihypertensive and anti-proteinuric effects (Dhakarwal et al., 2014). Parving et al. (2008), in their study, reported a daily dose of 300 mg of aliskiren causing a 20% reduction in the mean urinary albumin-to-creatinine ratio when compared to those who received a placebo. The blood pressure varied slightly among the treatment groups, with no difference between the total numbers of adverse and serious adverse events between the groups. This study suggested that aliskiren could be a renoprotective agent, with this attribute being independent of its blood-pressure-lowering effect in nephropathic patients (Parving et al., 2008). Comparable observations by Abe et al. (2013) also suggested the renoprotective effects of aliskiren in CKD independent of BP lowering. However, monotherapy of aliskiren in CKD is not extensively practiced and is less investigated, yielding inconclusive results (Abe et al., 2013). The focus has shifted more toward combination therapy with other antihypertensive agents. In various studies, combination therapy with aliskiren reported considerable reductions in blood pressure and proteinuria when compared with monotherapy in CKD (Wu et al., 2014). The trials that were undertaken to investigate the effect of aliskiren also have contradicting conclusions. The ALTITUDE trial attempted to evaluate the efficiency of aliskiren in diabetic patients at high risk for renal and cardiovascular events and concluded that patients had no benefit from aliskiren therapy but experienced increased renal complications with aliskiren treatment along with standard antihypertensive therapy. This was contrary to the ACCELERATE trial that yielded positive results for aliskiren use in combination with a calcium-channel blocker and was approved by FDA as a combination therapy; however, the recommendation did not include an ACEI or ARB (Guha et al., 2012). The DRINK (2021) study also declared aliskiren to have no additional benefit for renoprotection or increase in adverse events in non-diabetic CKD patients, except for more hyperkalemia events (Tang et al., 2021). All this evidence suggests that, for now, aliskiren does not have enough ground to be used as a first-line monotherapy for CKD.

ACEIs are regularly prescribed to patients with chronic kidney diseases. When introduced first in 1981, they were indicated only for the treatment of refractory hypertension but have since then been effective in improving the treatment outcome in various diseases, including chronic renal insufficiency (Bicket, 2002). They are similar in their mode of action, however, they differ in their metabolism, but this difference, if leading to one ACE being better than the other, has not been determined yet. In addition, the choice of the type of ACEI prescribed depends solely on the condition of the patient and the experience of the healthcare provider (Pugh et al., 2019). However, there are data suggesting renoprotection conferred by ACEI treatment in CKD and protection in nephropathies irrespective of their type (Bernadet-Monrozies et al., 2002). Moreover, this is more pertinent to CKD in its initial stages, and ACEIs seem to have no benefit or no adverse outcomes in advanced stages. Furthermore, in a real-world cohort, discontinuation of ACEI is common, especially in patients with lower eGFR (Qiao et al., 2019; Burnier, 2020). Bakris (2000) reported ACEIs to be more efficacious in decelerating the progression to end-stage renal disease in non-diabetic patients presenting with nephropathy when compared to other antihypertensives (Bakris, 2000). A meta-analysis by Xie et al. (2016) used randomized clinical trials and reported ACE inhibitors to be highly affiliated with probabilities of lowering kidney failure and reducing the risk of all-cause mortality when compared to ARBs. Studies such as angiotensin-converting enzyme inhibition in progressive renal insufficiency (AIPRI), ramipril efficacy in nephropathy (REIN), and EUCLID also demonstrated a decreased progression of renal disease with ACEI treatment (Maschio et al., 1999; Bicket, 2002). However, in diabetic nephropathy (type 2), there are not enough long-term trials to evaluate hard endpoints, especially progression to end-stage renal disease, with ACEI use; however, small trials with surrogate endpoints have yielded limited and conflicting results and the benefits of ACEIs in reducing the advancement to ESRD in type 2 diabetic patients is still not proven (Bakris and Weir, 2002). In normotensive patients having diabetic kidney disease (DKD), He et al. (2020), in their metanalytic study, also showed ACEIs lower albuminuria to varying levels with better response in patients with diabetes mellitus (He et al., 2020). On the other hand, Cai et al. (2018) reported individual ACEIs to have no or little benefit at goal doses on major renal outcomes in the diabetic kidney. In patients with renal transplants, Knoll et al. (2016), in their randomized trial, reported that ACEIs showed no association with improved clinical outcomes in the diabetic population. Paoletti et al. (2013) also observed that in renal transplant patients receiving ACEI, better survival free of the combined endpoint (death, cardiovascular events, loss of renal graft, or creatinine doubling) was observed without significant differences in renal outcome when compared to the controls (Paoletti et al., 2013). ACE inhibition is also associated with acute kidney injury and acute renal failure, which can be reversed by dose adjustments or withdrawal of the drug (Navis et al., 1996; Chaumont et al., 2016).

Most of the studies discuss ARBs along with ACEIs in CKD, indicating a similar outcome of therapy. ARBs have effects similar to ACEIs and are generally prescribed for patients unable to tolerate ACEIs (WEBMED, 2022). ARBs delay the progression of proteinuria both over short and long terms and to a similar level as ACEIs (Kunz et al., 2008). Burnier (2020) reported ARBS to be more specific in blocking the RAAS and having better tolerability when compared with ACE inhibitors (Burnier, 2020). ARBs have no detrimental effect on renal functions, and hence, the rate of discontinuation with ARBs is low, owing to their safety profile. Among the five major classes of antihypertensive drugs, the highest one-year compliance rate was observed for ARBs. ARBs present beneficial effects beyond controlling blood pressure and have renoprotective effects in diabetic nephropathy (Israili, 2000). In CKD patients with a non-dipping BP pattern, better renal protection was obtained with ARB treatment (Kunz et al., 2008). However, in patients with arterial blood pressure or renal function highly reliant on the RAAS, they can cause hypotension and/or renal failure; hence, they are inadvisable in patients with heart failure presenting with hypotension or bilateral renal artery stenosis (Hill and Vaidya, 2020).

Patients with mild or moderate chronic kidney disease have reliable improved outcomes with RAAS inhibitors, but less evidence supports their use in advanced CKD. The recently concluded STOP ACEI trial, which aimed to identify whether discontinuation of ACEIs/ARBs can improve or stabilize renal function in patients with advanced progressive CKD, reported that in such patients, the discontinuation of RAAS inhibitors was not observed to have a significant difference between the groups in the long-term rate of decrease in the eGFR. ESRD or the initiation of renal-replacement therapy was reported in 62% and 56% of the discontinuation group and the continuation group, respectively, and adverse events were similar in the discontinuation group and continuation group with respect to cardiovascular events (Bhandari et al., 2022).

## 9 Optimization of RAAS inhibitors

The RAAS inhibitors are the recommended first line of treatment for CKD but are associated with limitations that call for optimization with other pharmacologic interventions. Hyperkalemia is one such limitation that is of low occurrence in uncomplicated hypertension, but the incidence increases in the settings of other co-comorbidities or dual RAAS inhibition. Hyperkalemia is associated with an increased risk of death in patients with and without CKD (Weir and Rolfe, 2010; Lazich and Bakris, 2014; Pitt and Bakris, 2015). Treatment options for chronic hyperkalemia generally include withdrawal or dose reduction of RAAS inhibitors. This approach is undesirable since it withholds a life-saving or kidney-preserving therapy (Sterns et al., 2010). The initial management of hyperkalemia may also include enhanced urinary excretion of K by the use of loop diuretics, either alone or in combination with thiazide diuretics; however, this therapy is less effective in patients with advanced CKD or end-stage renal disease. Earlier pharmacological management of hyperkalemia used sodium polystyrene sulfonate (SPS) and calcium polystyrene sulfonate (CPS), cation-exchange resins, with no trials to ascertain their efficacy and safety. These older generation K^+^ binders were contraindicated in CKD associated with hypercalcemia or vascular calcification, leading to poor adherence (Valdivielso et al., 2021). Recently, two new K^+^-binding drugs have been approved by FDA for the treatment of hyperkalemia: sodium zirconium cyclosilicate (SZC) and patiromer. SZC exchanges K^+^ for sodium and hydrogen ions in the gastrointestinal tract, while patiromer exchanges K^+^ for calcium ions in the gastrointestinal tract. These new potassium binders may present greater selectivity for potassium than other cations, hence resulting in a lower risk of clinical electrolyte abnormalities (Natale et al., 2020). Potassium binders should never be the exclusive treatment regimen of hyperkalemic emergencies, but the combination therapy of the K^+^ binders and RAAS inhibitors is expected to improve the treatment outcomes in CKD by allowing continued use of RAAS inhibitors without developing hyperkalemia (Tinawi, 2020). Also, an affirmatory safety profile comes as an advantage of the K^+^ binders. Studies such as AMETHYST-DN 2015; HARMONIZE 2014; OPAL-HK, and Packham et al. (2015); PEARL-HF 2011; and the studies by Weir and Rolfe (2010) have strongly indicated newer K^+^ binders lower and maintain serum potassium levels in CKD patients prescribed RAAS inhibitors (Kosiborod et al., 2014; Bakris et al., 2015; Ali et al., 2020). Patients receiving RAAS inhibitor therapy and patients with severe hyperkalemia had consistent reductions in serum K^+^ with SZC (Pitt et al., 2011; Ash et al., 2015). In CKD patients with hyperkalemia or risk of developing hyperkalemia, patiromer significantly reduced serum K^+^ concentrations, thereby facilitating the continuation of RAAS inhibitor therapy (Valdivielso et al., 2021).

Another presentation that requires optimization is diabetic kidney disease. DKD is a leading cause of CKD, with half of the patients entering end-stage renal disease. Treatment of CKD in diabetes takes effort as patients with diabetic kidney disease present microvascular complications and are at very high risk for developing macrovascular complications. The first line of treatment is to slow or prevent the decline in GFR and prevent further microvascular and macrovascular complications. One of the most critical primary preventive measures is glycemic control maintaining A1C < 6.5%, followed by stringent BP control (DeFronzo and Abdul-Ghani, 2021). A limited choice of glucose-lowering agents was available for diabetic patients with CKD arising out of safety issues, adverse effects, or lack of evidence in people with low glomerular filtration rates. Sodium-glucose cotransporters type 2 inhibitors (SGLT2 inhibitors) came up as novel agents for the management of type 2 diabetes mellitus. The use of SGLT2 inhibitors has led to a sturdy A1c reduction of 0.5%–0.6%, sustained over 52 weeks of follow-up, and have a range of effects that lead to valuable benefits beyond glycemic control, including BP and weight reduction (Monami et al., 2014). In addition to glycemic control, restoration of tubuloglomerular feedback is a mechanism that can account for the beneficial kidney effects of SGLT2 inhibitors Cherney. For diabetic patients with CKD, SGLT2 inhibitors modestly lower A1C and fasting plasma glucose and are associated with a substantial reduction in albuminuria and reduced risk of progression to albuminuria Kelly. Combination therapy with SGLT2 inhibitors and ACEIs/ARBs in T2DM is known to be more effective and well-tolerated than ACEI/ARB alone and yielded better treatment outcomes in terms of control of blood pressure, improvement of renal outcomes, improvement of long-term renal function, and a decrease in blood glucose and body weight Tian B. In non-diabetic patients with albuminuric CKD, treatment with the combination of ACEIs/ARBs and SGLT2 inhibitor significantly increased kidney failure-free survival (Vart et al., 2022). Trials such as EMPA-REG OUTCOME, CREDENCE, and DAPA-CKD have given encouraging results. SGLT2 inhibitors seem to have their beneficial effects independent of their blood glucose-lowering effects and possibly mediated by natriuresis and glucose-induced osmotic diuresis, which leads to a reduction in intraglomerular pressure. Persons with kidney diseases due to causes other than type 2 diabetes may also have their kidney function preserved by this hemodynamic effect (Heerspink et al., 2018; Cherney et al., 2020; van Bommel et al., 2020). In patients with CKD presenting with hyperkalemia where up-titration of RAAS blockade may be prohibited, SGLT2 inhibitors may enhance kaliuresis by increasing distal delivery of sodium and stimulating aldosterone, thereby reducing hyperkalemia (Yau et al., 2022). In CREDENCE, which included patients with T2DM and CKD on RAAS blockade, canagliflozin reduced the incidence of hyperkalemia by 23% without causing hypokalemia (Neuen et al., 2021). There is a comparable glucose-lowering efficacy and safety of SGLT2 inhibitors in patients with mild CKD as in patients with normal kidney function. Patients with moderate CKD may have the efficacy suppressed, and safety concerns may occur, while in patients with severe CKD, the use of SGLT2 inhibitors is contraindicated. Hence, care and caution need to be exercised before prescribing SGLT2 inhibitors (Scheen, 2015).

Patients with CKD also have a higher incidence of cardiovascular events, both acute and chronic, which, in turn, increase the risk of progression to ESRD. In such cases, inhibition of neprilysin could present a potential improvement strategy in the cardiovascular and renal outcomes of patients with CKD (Judge and Landray, 2015). The concept of blocking neprilysin is not very recent, but the drugs used earlier as neprilysin inhibitors had an unacceptable incidence of angioedema (Di Lullo et al., 2017). Neprilysin inhibitors were initially combined with ACEIs, forming vasopeptidase inhibitors (VPIs). Omapatrilat was the most widely studied VPI, which showed initial promising results but was associated with unacceptable rates of angioedema and was later withdrawn (Judge and Landray, 2015). Neprilysin inhibition (NEPI) enhances the activity of natriuretic peptide systems causing subsequent natriuresis, diuresis, and inhibition of the RAAS, acting as a significantly beneficial counter-regulatory system in conditions of RAAS activation such as chronic heart failure and CKD. The latest generation molecules developed can inhibit the neprilysin receptor and the angiotensin II receptor simultaneously and are known as angiotensin receptor neprilysin inhibitors (ARNIs). The first angiotensin receptor neprilysin inhibitor to be produced combines an ARB (valsartan) and a NEPI prodrug (sacubitril) in a 1:1-M complex and has shown significant delay in the progression of CKD (Judge and Haynes, 2021). Increasing evidence strongly suggests that sacubitril/valsartan is superior to conventional RAAS inhibitors in lowering blood pressure in patients with hypertension; however, patients must be able to tolerate ACEI or ARB before being started on sacubitril/valsartan (Vicent et al., 2019; Yamamotoand Rakugi, 2021). Worst renal outcomes were associated with increase in albuminuria with enalapril therapy; an increase in the ACR was not related to worse renal outcome with ARNI therapy (Damman et al., 2018). The PARADIGM-HF study has reported a reduction in diuretic need in the ARNI group, indicating a decrease in the progression of CKD (Cho and Kang, 2021). The PARAMOUNT trial reported a slower deterioration of eGFR in patients with HFpEF after sacubitril/valsartan use (Tersalvi et al., 2020).

Even though RAAS inhibitors are paramount in the treatment of CKD, many gaps need to be filled to obtain the best possible treatment outcomes, and combination therapies are used and being developed to fill in the voids that remain with RAAS inhibition therapy.

## 10 Influence of gene polymorphisms on response to RAAS inhibition

Hypertension is a feature of about 80% of CKD patients, and the current guidelines indicate the pharmacologic blockade of the RAAS as the first choice of antihypertensive intervention. However, both response to treatment and renal protection show significant inter-individual variability (Santos et al., 2012). The variability may arise due to varying levels of ACE in plasma and tissue being partly determined by a genetic polymorphism. This is more common in Asian people having the DD genotype, showing increased activity of ACE, and hence are at higher risk for nephropathy (Rudnicki and Mayer, 2009). A total of 44 genes in the Pharmacogenomics Knowledge Database are considered highly significant pharmacogenes for their influence on the function and diseases of the kidney (Bochud et al., 2011). The symbolic genes involved in CKD disease are the CYP1A2 and CYP3A5, ABCB1, and methylenetetrahydrofolate reductase (Padullés et al., 2014). Very limited data are available on the polymorphic variations and pharmacogenomics of RAAS inhibitors specifically for outcomes in CKD, to the best of our knowledge, and further research on this perspective can lead to improved life expectancy and quality of CKD patients.

To evaluate the effect of genetic variations on the pharmacokinetics and pharmacodynamics of aliskiren, Tapaninen et al. (2010) studied the outcomes of common haplotypes of the ABCB1 gene encoding P-gp, c.1236C-c.2677G-c.3435C and c.1236T-c.2677T-c.3435T and the effects of c.935G>A single-nucleotide polymorphism (SNP) in the SLCO2B1 gene encoding OATP2B1. However, no significant association between haplotype or SNP and the pharmacokinetics or pharmacodynamics of aliskiren was observed. An increase *in vitro* renin gene transcription was reported with a T allele variant (position−5312) occurring within a distal enhancer region. *In vivo* functional activity with aliskiren is reported for renin −5312C/T, with lesser reductions in BP observed in −5312T allele carriers than in CC homozygotes (Moore et al., 2007).

For ACEIs and ARBs, there was no influence of CYP3A5*3 on pharmacogenetics in CKD (Lee et al., 2021).

## 11 Pricing and global market

The cost of the treatment is a significant factor in determining patient compliance and persistence for a drug. The price determines which RAAS inhibitor can be brought into the day-to-day routine of patient management. The cost of the drug influences the treatment practices that rely on intraclass pharmacologic differences (Sica et al., 2000).

Aliskiren has a market segmented by country, players, type, and application. The brands are more expensive than other similar medications; however, lower cost generics are available (Tekturna, 2022). The daily cost of an aliskiren is $ 1.14, which is higher than that of other antihypertensives (CADTH, 2022). It is estimated to have a cost-effectiveness of $30,500 per quality-adjusted life year gained for ESRD in T2DM (Delea et al., 2009). The aliskiren market size has grown at a moderate pace and is expected to grow significantly in the period 2021–2028 (Verified Market Research, 2022).

ACEIs have variable cost and availability depending on region- and institution-specific purchasing contracts (Gerbrandt and Yedinak, 1996). Various cost-effectiveness models have demonstrated ACE inhibitor treatment in CKD to be cost-effective (Schädlich et al., 2001; Adarkwah et al., 2013). An estimated value of US$ 11,693.6 million was provided for the global market of ACE inhibitors in 2017, with an expected 0.8% CAGR for the forecast period of 2018–2026 (Angiotensin Converting Enzymes, 2022). Treatment of diabetics with ACEIs was less costly (average lifetime saving of $A825 per patient) than without ACEIs. Generic forms of certain ACEIs are available that cost less (Howard et al., 2010). In autosomal dominant polycystic kidney disease, which can cause CKD, the annual healthcare cost for taking ACEIs was estimated to be $3,505,028.41, with an increase of 1.39 years in the life expectancy of the patients.

ARBs are more expensive than ACEIs and are comparable to aliskiren ($1.02–$1.36 cost per day); however, the generic availability of ARBs enables them to be included in insurance formularies. In autosomal dominant polycystic kidney disease, ARBs incurred an annual healthcare cost of $3,644,327.65, with an approximate 10-year survival of ARB at 34% compared to 47% for ACEIs (Clark et al., 2017). The value of the global market for ARBs was USD 7.85 billion in 2020 and is predicted to reach USD 9.95 billion by 2028, increasing at a CAGR of 3.4% from the period 2021 to 2028 (Verified Market Research, 2022).

## 12 Conclusion

The RAAS inhibitors are the preferred class of drugs recommended for the treatment of CKD; however, they slow down the progression of CKD by ∼20% compared with other therapies, instead of stopping its progression. The RAAS inhibitors have a wide array of structures, but their attribute to inhibit the RAAS places them on a comparable platform. Their mechanisms of action also vary with the DRI reckoned to overcome the side effects of ACEIs and ARBs. Amongst the RAAS inhibitors, ACEIs and ARBs seem to have a profound effect on treatment outcomes in CKD when compared to DRI. The ACEIs and ARBs are comparable in most of their attributes, but ARBs have an edge in the patients intolerant to ACEIs. The pricing favors the use of ACEIs to maintain sustained use. A direct comparison becomes difficult owing to differences in structure, mechanism of action, design model, region settings, etc., in the three groups of RAAS inhibitors, and this implies that drugs should be prescribed considering the balance of benefit and risk required by the individual. The drugs, however, face a challenge on account of new therapies targeting RAAS with higher renal protection conferred in comparison to DRI, ACEIs, and ARBs.
